# Shared Leadership and Team Effectiveness: An Investigation of Whether and When in Engineering Design Teams

**DOI:** 10.3389/fpsyg.2020.569198

**Published:** 2021-01-18

**Authors:** Qiong Wu, Kathryn Cormican

**Affiliations:** ^1^School of Business, Macau University of Science and Technology, Macau, China; ^2^Lero – The Irish Software Research Centre, School of Engineering, National University of Ireland, Galway, Ireland

**Keywords:** shared leadership, team effectiveness, project life cycle, social network analysis, engineering design teams

## Abstract

Shared leadership is lauded to be a performance-enhancing approach with applications in many management domains. It is conceptualized as a dynamic team process as it evolves over time. However, it is surprising to find that there are no studies that have examined its temporally relevant boundary conditions for the effectiveness of the team. Contributing to an advanced understanding of the mechanism of shared leadership in engineering design teams, this research aims to investigate *whether* shared leadership is positively related to team effectiveness and *when* shared leadership is more likely to be effective. Using a field sample of 119 individuals in 26 engineering design teams from China and the technique of social network analysis, we found that, consistent with cognate studies, shared leadership is positively related to team effectiveness when measured in terms of team task performance and team viability. Moreover, by integrating the project life cycle as a moderator, this study is among the first to investigate the temporal factors, for the effectiveness of shared leadership. The result shows that the stage of the project life cycle moderates the positive shared leadership-team effectiveness relationship, such that this association is stronger at the early phase than at the later phase of the project. Overall, these findings offer insightful thoughts to scholars in the field of shared leadership and bring practical suggestions for project managers in business who seek to implement best practice in organizations toward high team effectiveness.

## Introduction

In recent years, leadership researchers have emphasized a team-level phenomenon, where leadership is carried out by the team as a whole, rather than exclusively by those at the top or by those in formal leadership positions (Carson et al., 2007; Pearce et al., 2014). As such, the notion of shared leadership has gained more traction in the extant literature. By definition, shared leadership is described as “a dynamic, interactive influence process among individuals in groups for which the objective is to lead one another to the achievement of group or organizational goals or both” (Pearce and Conger, 2003, p. 1). As Acar (2010) noted, shared leadership represents a fundamental shift away from the notion of a single, appointed leader, to the idea that team members mutually influence each other and collectively share leadership roles, responsibilities and functions. Recent empirical work has provided evidence for the important role of shared leadership in groups (Nielsen and Daniels, 2012; Nicolaides et al., 2014; Sousa and Van Dierendonck, 2016; Sun et al., 2016). More interestingly, some studies have even found that shared leadership is more influential than convectional vertical leadership for team effectiveness (Pearce and Sims, 2002; Ensley et al., 2006). However, our understanding of *whether* shared leadership is positively related to team effectiveness and *when* shared leadership is more likely to be effective is still limited in at least three fundamental ways.

First, in recent years, researchers and practitioners have advocated the benefits of shared leadership as a way to promote team effectiveness. For example, Ramthun and Matkin (2012) stated that shared leadership is often advantageous, since members are more likely to follow the person having the best knowledge and skills than depending solely on the vertical influence process of traditional leadership. Indeed, many other empirical studies have also demonstrated that teams with shared leadership yield higher team effectiveness (Pearce and Sims, 2002; Wang et al., 2014; Serban and Roberts, 2016). However, we must caution that this is not always the case. Fausing et al. (2013) and Mehra et al. (2006) failed to find support for this significant and positive relationship, and Boies et al. (2011) even found that shared leadership exerts a negative influence on team effectiveness. Such inconsistent findings point to the need for more empirical evidence. Therefore, in order to enrich our understanding of the value of shared leadership, the first purpose of our study is to explicitly examine the shared leadership – team effectiveness relationship. In this study, we define team effectiveness as the extent to which teams meet the expectations of organizations (Essens et al., 2009). This viewpoint encourages us to think about team effectiveness from a multidimensional perspective. Consequently, we follow Aube and Rousseau (2005), Balkundi and Harrison (2006), and Mathieu et al. (2008), who consider team effectiveness from two distinct aspects: team task performance and team viability. Team task performance refers to how well the group meets (or even exceeds) work expectations while team viability is the potential of teams to retain its members and to function effectively over time (Balkundi and Harrison, 2006).

Second, in order to gain a more fine-grained understanding of the impacts of shared leadership, unanswered questions must be addressed. More specifically, there is a clear need to investigate the temporally relevant moderators for its effectiveness. Researchers have emphasized that shared leadership is a dynamic, emergent, time-varying construct (Avolio et al., 2009) that is affected by the environment of a team (Carson et al., 2007; Wu et al., 2020) and task characteristics (Serban and Roberts, 2016; Hans and Gupta, 2018). Therefore, continuous changes in the inputs, processes and outputs of different phases of the project life cycle could influence the emergence of shared leadership in teams (Wu and Cormican, 2016) as well as its relationship with team effectiveness. However, the potential moderating impact of the project life cycle for the effectiveness of shared leadership is not well theoretically developed nor rigorously empirically tested. This important unaddressed gap needs further attention so as to provide insights into the boundary conditions regarding when shared leadership is more or less influential to team effectiveness. Consequently, the second research goal is to focus on the dynamic nature of shared leadership and investigate the moderating effect of the project life cycle in the relationship between shared leadership and team effectiveness.

Third, although there is growing interest in the shared leadership domain, studies concentrating on project teams are still limited and under-developed (Scott-Young et al., 2019). Shared leadership theory has been widely spread and applied across a range of team types, e.g., top management teams (Singh et al., 2019), entrepreneurial teams (Zhou, 2016), consulting teams (Carson et al., 2007), and change management teams (Pearce and Sims, 2002). However, there is a dearth of investigations relating to project teams. While the current workplace is becoming increasingly project-centric (Scott-Young et al., 2019), there remain very few studies focusing on shared leadership theory in the project management context. In order to extend the external validity of the shared leadership construct in project settings, this study examines the effectiveness of shared leadership in project-based engineering design teams. Moreover, as project teams uniquely have definitive start and end times based on the duration of the tasks (Farh et al., 2010), it is well suited to help explain when shared leadership is more likely to be effective in teams.

Taken together, this research seeks to enrich our understanding of the mechanisms of shared leadership and investigates *whether* and *when* shared leadership is positively related to team effectiveness in engineering design teams. To do this, we used the social network approach to measure the construct of shared leadership by calculating network density and creating binary matrices as well as sociograms. Team effectiveness was measured using nine items consisting of two separate, theoretically derived subscales: *team task performance* and *team viability*. Moreover, an internal consistency analysis and confirmatory factor analysis was performed to assess the reliability and validity of our measurement model. We then conducted a two-way moderated hierarchical regression analysis (Carson et al., 2007; Erkutlu, 2012; Fausing et al., 2013) in this study so as to test hypotheses proposed. By doing so, our study makes several significant contributions: (1) it extends a line of research and explicitly examines the relationship between shared leadership and team effectiveness; (2) it builds on the dynamic nature of shared leadership and is among the first to investigate an important temporal moderator, the project life cycle, for the effectiveness of shared leadership; (3) it adds to the academic debate by extending the external validity of shared leadership theory in engineering design teams; (4) it brings insightful thoughts to the field of project management by providing practical suggestions for project managers in business who seek to implement best practice in their organizations.

## Theory and Hypotheses

### Shared Leadership Theory

Leadership scholars have realized the importance of shared leadership and worked to understand how to conceptualize it, measure it, and to assess what impacts it brings to teams. Table 1 presents details of relevant prior empirical studies. As illustrated, conceptually, shared leadership is a team-centric phenomenon (Ensley et al., 2006; Serban and Roberts, 2016) whereby team members engage in “leadership roles and responsibilities on behalf of the team” (Robert and You, 2018, p. 503), and “accepts their colleagues’ leadership” (Aubé et al., 2017, p. 199). Furthermore, shared leadership is not a static process; it is defined as an emergent, dynamic phenomenon that unfolds over time (Avolio et al., 2009; Drescher et al., 2014; Wang et al., 2014). According to Carson et al. (2007), shared leadership is considered in terms of a continuum ranging from low to high, which implies that shared leadership is not a rigid either-or category, but occurs in every group at various levels (Liu et al., 2014).

**TABLE 1:** Definitions, measures, and impacts of shared leadership.

Studies	Definitions	Measures	Contexts	Countries of sample	Dependent variables
Pearce and Sims (2002)	A group process in which leadership is distributed among, and stems from, team members (p. 172).	Aggregation	Change management teams	United States	Team effectiveness (self-reported and manager ratings)
Sivasubramaniam et al. (2002)	Collective influence of members in a team on each other (p. 68).	Aggregation	Undergraduate student teams	United States	Team performance (self-reported)
Ensley et al. (2006)	A team process where leadership is carried out by the team as a whole, rather than solely by a single designated individual (p. 220).	Aggregation	Top management teams	America	Team performance (objective indicators)
Mehra et al. (2006)	Shared, distributed phenomenon in which there can be several (formally appointed and/or emergent) leaders (p. 233).	Social network analysis	Financial service sales teams	United States	Team performance (self-reported and objective indicators)
Carson et al. (2007)	An emergent team property that results from the distribution of leadership influence across multiple team members (p. 1218).	Social network analysis	Consulting teams (MBA students)	United States	Team performance (external ratings)
Acar (2010)	The sharing of leadership roles, responsibilities, and functions among all group members (p. 1740).	Aggregation	Students teams	United States	Diversity and emotional conflict (self-reported)
Bergman et al. (2012)	The number of members on the team who performed positive leadership behaviors; and the amount of leadership behavior exhibited by the team (p. 26).	Social network analysis	Decision making teams (undergraduate students)	United States	Team Functioning (self-reported)
Erkutlu (2012)	Serial emergence of temporary leaders, depending on the tasks facing the team and the knowledge, skills and abilities of the team members (p. 104).	Aggregation	Commercial bank teams	Turkey	Team proactive behavior (self-reported)
Drescher et al. (2014)	An emergent property of a group where leadership functions are distributed among group members (p. 772).	Aggregation	Strategy game teams	Worldwide	Team performance (objective indicators)
Liu et al. (2014)	Involves non-hierarchical relationships and describes a relational phenomenon that is characterized with a dynamic, interactive influence process among individuals in the team (p. 284).	Social network analysis	Work teams	China	Team and individual learning
Lee et al. (2015)	A voluntarily, informally emergent structure beyond vertical leadership (p. 47).	Social network analysis	E-learning teams (undergraduate students)	South Korea	Team creativity (self-reported)
Serban and Roberts (2016)	A team-based collective phenomenon (p. 182); The actions and decisions of a team are not the result of a single leader acting toward the team, but of the team itself (p. 181).	Social network analysis	Student teams	England	Task and team satisfaction, team performance (self-reported)
Chiu et al. (2016)	Emended in interaction among team members (p. 1707).	Social network analysis	Work teams	China	Team performance (manager ratings)
Aubé et al. (2017)	Each team member engages in leadership functions and accepts their colleagues’ leadership (p. 199).	Social network analysis	Project teams (students)	Canada	Teamwork behaviors (self-reported)
Robert and You (2018)	The degree to which the typical team member engages in leadership roles and responsibilities on behalf of the team (p. 503)	Social network analysis	Virtual teams (students)	United States	Team members’ trust, autonomy, satisfaction (self-reported)

While progress has been made relating to the definitions of shared leadership, many empirical studies have centered on what impacts shared leadership brings. As shown in Table 1, the positive relationship between shared leadership and team performance has received much attention (Sivasubramaniam et al., 2002; Ensley et al., 2006; Mehra et al., 2006; Carson et al., 2007; Drescher et al., 2014). Additionally, shared leadership is also demonstrated to be positively related to team functioning (Bergman et al., 2012), team proactive behavior (Erkutlu, 2012), team and individual learning (Liu et al., 2014), team member’ diversity and emotional conflict (Acar, 2010), team members’ trust, autonomy and satisfaction (Robert and You, 2018). These findings are encouraging and suggest the need for more sophisticated designs on the notion of shared leadership. Accordingly, this study extends a line of research to further examine its relationship with team effectiveness and goes beyond simple relationships to investigate when shared leadership plays a stronger or weaker role in the effectiveness of teams. The relevant research hypotheses are proposed below.

### Shared Leadership and Team Effectiveness

Based on the work of Aube and Rousseau (2005), Balkundi and Harrison (2006), and Mathieu et al. (2008), team effectiveness is considered in terms of two distinct aspects: team task performance (how well the group meets (or even exceeds) work expectations) and team viability (the potential of teams to retain its members and to function effectively over time). This assessment conforms to the classic work of Barrick et al. (1998), who suggested that a comprehensive assessment of team effectiveness should capture both current team effectiveness (i.e., present task performance) and future team effectiveness (i.e., capability to continue working together). Therefore, this research adopts a broad perspective to team effectiveness and explores the relationship between shared leadership and team effectiveness.

First of all, this study expects that shared leadership is positively associated with team task performance. As suggested by Day et al. (2004), shared leadership advances the social capital of the team *via* the utilization of team resources such as the knowledge and capability of group members, which subsequently fosters team task performance. Katz and Kahn (1978) also proposed that when group members offer leadership to others and to the mission or purpose of their group, they bring more personal and organizational resources to the task, share more information, and they experience greater commitment. Further, when group members are influenced by their fellows, team functioning is improved as high levels of respect and trust are evidenced among group members. Collectively, teams exhibiting these characteristics, can also exhibit greater levels of performance (Day et al., 2004). This premise aligns with many empirical studies (see Table 1). For instance, Carson et al. (2007), in a study of 59 consulting teams, found that shared leadership is positively associated with team performance as rated by clients. Ensley et al. (2006), in a study of 66 top management teams, demonstrated that shared leadership is a more significant predictor than vertical leadership of new venture performance when considered in terms of revenue and employee growth. Furthermore, Drescher et al. (2014), in a longitudinal examination of 142 teams who engaged in a strategic simulation game, also demonstrated support for the positive influence of shared leadership on team task performance. Taken these together, this study proposes:

*Hypothesis 1a:* Shared leadership is positively related to team task performance in engineering design teams.

Shared leadership, as an important intangible resource available to teams (Carson et al., 2007), fosters not only team task performance, but also team viability. As Wood and Fields (2007) suggested, shared leadership exerts a series of positive impacts on team members’ job perceptions: it brings low levels of role overload, role conflict, role ambiguity and job stress, as well as high levels of job satisfaction. Similarly, Bergman et al. (2012) also demonstrated that teams with shared leadership experience less conflict, greater consensus, and higher intragroup trust and cohesion. This may foster team viability as members in shared leadership teams experience increased interdependence, more collaboration, and they sense greater levels of satisfaction. Additionally, when there is effective coordination and collaboration among team members fulfilling leadership responsibilities, it is easier for them to identify the potential causes of conflicts and propose potential solutions. It thus reduces the amount of conflict and promotes team consensus and trust (Balkundi and Harrison, 2006). As a consequence, team viability, which retains members and maintains good team functioning over time, could be enhanced. This research therefore posits:

*Hypothesis 1b:* Shared leadership is positively related to team viability in engineering design teams.

Taken these two hypotheses (hypothesis 1a and 1b) together, this study expects that shared leadership will foster team effectiveness by enhancing team task performance and team viability. As Wang et al. (2014) suggested, shared leadership nurtures a collective identity among members of the team and strengthens the level of engagement with and commitment to the group, which in turn enhances team effectiveness. Moreover, Mathieu et al. (2015) mentioned that shared leadership fosters social inclusion and enhances team cohesion, which can, subsequently, facilitate team effectiveness. In light of this, this research suggests:

*Hypothesis 1c:* Shared leadership is positively related to team effectiveness in engineering design teams.

### The Moderating Role of the Project Life Cycle

Notwithstanding the fact that research on the relationship between shared leadership and team effectiveness brings valuable insights into the understanding of shared leadership in teams, there is an important omission in prior studies regarding its temporal moderating roles on such a relationship (Carson et al., 2007; D’Innocenzo et al., 2014; Wang et al., 2014). In an attempt to open the black box, this study seeks to examine a potential moderator of shared leadership, namely the project life cycle, and expects that the positive association between shared leadership and team effectiveness will be stronger at the early phase than the later phase of the project. This is because the focal concern of the early stage is toward planning and strategy generation (Chang et al., 2003; Farh et al., 2010), where project team members are more willing to engage in mutual leadership as they become proactively involved in constructive communication and decision-making (Wu and Cormican, 2016). It thus allows individuals to bring more resources to the task, share more information, and to experience higher levels of commitment (Bergman et al., 2012). Collectively, these consequences would result in greater team effectiveness (Day et al., 2004; D’Innocenzo et al., 2014). Furthermore, as time and resources are less constrained at the early stage (Farh et al., 2010), members are able to take initiative to develop their own leadership abilities as well as to facilitate the leadership skills of others, which subsequently fosters the effectiveness of project teams (Ensley et al., 2006; Serban and Roberts, 2016). However, when the project advances into the later stage, resources are dedicated to execute project plans (Farh et al., 2010). This leads to a change in the leadership distribution from many team members to a few individuals, who assume the responsibility of integrating resources, controlling the development of the project to meet deadlines and keeping costs within budget (Wu and Cormican, 2016). Teams may no longer afford to spend too much time cultivating a positive team environment to promote shared leadership (Carson et al., 2007). As such, any potential of shared leadership for enhancing team effectiveness would be more difficult to realize in the later stage of the project life cycle. Therefore, this research expects that:

*Hypothesis 2*: The stage of the project life cycle moderates the positive association between shared leadership and team effectiveness, such that this relationship will be stronger at the early phase than at the later phase of the project in engineering design teams.

## Methodology

### Research Setting and Sample

A survey-based design was conducted in this study. The sample comprised 26 project-based engineering design teams working in the construction industry in China. As suggested by Carson et al. (2007), shared leadership is effective for teams composed of knowledge-based employees, because people having high levels of expertise and skills seek autonomy in how they apply their specialties, and thus desire more opportunities to shape and participate in the leadership functions for their groups. Engineering design teams comprising knowledge workers have the potential to leverage the expertise of a diverse group of members by pooling their talent and knowledge. This kind of team is likely to nourish the emergence or development of shared leadership. This perspective thus adds to the academic debate on the relationship between shared leadership and team effectiveness and extends the external validity of shared leadership theory into engineering design teams. Moreover, we chose a Chinese sample due to the fact that the conceptualization and operationalization of shared leadership is predominantly developed in the Western countries (see Table 1) and it remains uncertain whether its theoretical models hold up in Chinese cultural settings. Furthermore, scholars, like Whetten (2009), have called for more attention to be paid to explaining cultural context effects. Therefore, to plug this gap, this study seeks to extend the validity of the shared leadership construct to a Chinese context, whereby its organizational culture differs from Western countries. Specifically, according to Hofstede et al. (2005), the power distance and collectivism in China are rated stronger than in Western cultures. Initially, a pilot test was conducted with 16 employees from three engineering design teams. Based on feedback provided, minor modifications to the survey items were made. Next, 146 members from 34 engineering design teams were invited to participate in this study. Of the 146 participants who received the questionnaire, 127 returned it, yielding an 87% response rate. Teams with less than three members were eliminated from the sample. It resulted in a sample of 119 employees working in 26 project teams. The average team size of the sample is 5.26. The specific participant demographics are outlined in the Table 2.

**TABLE 2:** Sample characteristics.

Characteristics	Frequency	Percentage	Characteristics	Frequency	Percentage
**Age (years old)**			**Highest education**		
< = 20	0	0	High school degree or equivalent	2	2%
21–30	57	48%	College degree	76	64%
31–40	47	39%	Master’s degree	30	25%
41–50	9	8%	Doctoral degree	8	7%
More than 50	6	5%	Others	3	3%
**Gender**			**Role**		
Male	69	58%	Project manager	28	24%
Female	50	42%	Designer/planner	37	31%
			Engineer	26	22%
**Working experience (years)**			Operators	15	13%
< = 2	15	13%	Admin/supervision	7	6%
3–5	51	43%	Others	6	5%
6–10	38	32%			
> = 11	15	13%			
Total	119	100%		119	100%

### Measures

#### Shared Leadership

This research study adopted a social network approach to assess the nature of shared leadership. The social network technique is an intrinsically relational method that advocates a natural theoretical and analytical method to modeling the patterns of the relationships among interconnected individuals (D’Innocenzo et al., 2014). This study used the most common index of social network analysis, network density, to explicitly measure the extent to which team members are perceived to be involved in the sharing of leadership (Wang et al., 2014). This popular measurement was employed in many empirical studies of shared leadership (Carson et al., 2007; Lee et al., 2015; Chiu et al., 2016; Serban and Roberts, 2016). Following Carson et al. (2007), this study assessed the level of shared leadership by requiring every team member to rate each of his/her peers on the following question: “To what degree does your team rely on a particular individual for leadership?” A five-point Likert scale was used to measure the level of perceived leadership, where 1, represents “not at all,” and 5, “to a very great extent.” Network density was then calculated by summing all of the responses from group members divided by the total number of possible relations among group members (Carson et al., 2007; Mathieu et al., 2015). The values of density ranged from 0 to 1, where higher values indicate higher degrees of shared leadership within a team. Furthermore, as shared leadership is a team-level phenomenon, agreement among the respondents’ ratings of group members was also measured thus proving appropriate interrater reliability [mean *r*_wg_ = 0.75, ICC(1) = 0.44, ICC(2) = 0.77].

**FIGURE 1:**
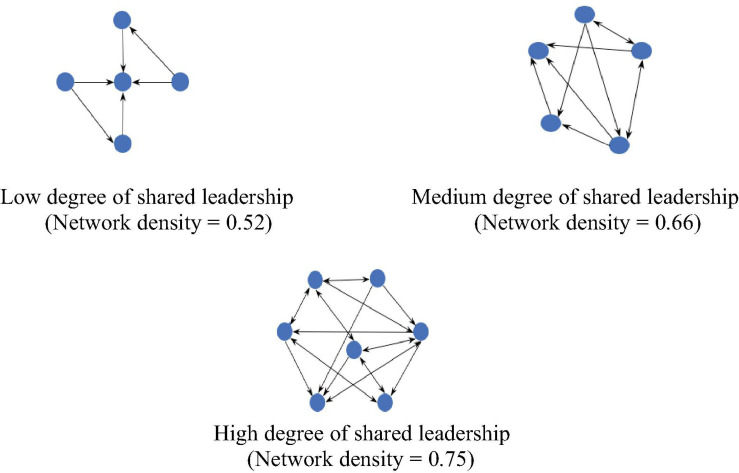
Leadership sociograms in this study. Low degree of shared leadership Medium degree of shared leadership (Network density = 0.52) (Network density = 0.66). High degree of shared leadership (Network density = 0.75).

To visually represent the density of shared leadership, this study developed leadership sociograms for each sample team similar to Carson et al. (2007) and Pastor and Mayo (2002). To do this, binary matrices were created, which were then used to quantify the degree of leadership influence for each team and to represent the presence or absence of leadership relations between pairs of team members. More specifically, the raw leadership ratings collected from each participant were aggregated and included in g × g squared matrices. These data were then dichotomized, where values of 4 (to a great extent) or 5 (to a very great extent) are considered as 1, and values of 3 and less are given a value of 0. The second step was to create leadership sociograms based on these binary matrices. Figure 1 shows the leadership sociograms in our study. Specifically, it illustrates three examples with low, middle and high levels of density of shared leadership networks. Among all of our sample data (26 engineering design teams), 0.52 is the lowest score, 0.66 is the medium score, and 0.75 is the highest score of network density. The nodes symbolize team members and the arrows represent leadership relations. One arrow points from team member (A) to member (B), indicating that B is perceived as a source of leadership by A. In this vein, two-headed arrows imply that two members perceive each other as a source of leadership.

#### Team Effectiveness

Team effectiveness was measured by team participants (including team leaders and members) *via* nine items consisting of two separate, theoretically derived subscales: *team task performance* and *team viability* using a five point Likert scale ranging from 1 “strongly disagree” to 5 “strongly agree.” Team task performance was assessed using five items derived from Sousa and Van Dierendonck (2016) and Suprapto et al. (2018). It measures the degree to which the project meets its goals, quality, schedule, budget, and overall level of customer satisfaction. Team viability was measured using four items derived from Aube and Rousseau (2005). These include the extent of a team’s capacity to solve problems, the ability to integrate new members, the ability to adapt to changes, as well as the ability to continue to work together in the future. In order to test for the discriminant validity, a confirmatory factor analysis (CFA) was performed. This yielded a good fit to the data (X^2^_27_ = 33.90, CFI = 0.99, GCI = 0.94, AGFI = 0.09, RMSEA = 0.05). These CFA results demonstrate the support for the hypothesized structure to measure team effectiveness. This study further examined the correlation between these two subscales to check the convergent validity of this measurement model. The finding provides evidence that these two subscales are highly correlated with each other (*r* = 0.92, *p* < 0.001). Given the strong support of the hypothesized measurement model, this study aggregated these two subscales to the group level and then averaged the scores to generate a single variable to represent team effectiveness (Cronbach α = 0.95). To justify whether this aggregation is appropriate, this research used the interrater agreement statistic, *r*_wg_ (James et al., 1993). The mean *r*_wg_ value of 0.82 was much larger than the conventional cut-off value of 0.70 (James et al., 1993), which implies that on average, there is a high degree of agreement among different raters with a group. Furthermore, the intraclass correlation coefficient, ICC (1) and the reliability of the group-level mean, ICC (2) were also calculated to test between-group variance and within-group agreement (Bliese, 2000). The results showed that the ICC (1) value of 0.73 suggested that team membership accounted for significant variance and the ICC (2) value of 0.92 demonstrated that the group-level means were reliable.

#### Project Life Cycle

**FIGURE 2:**
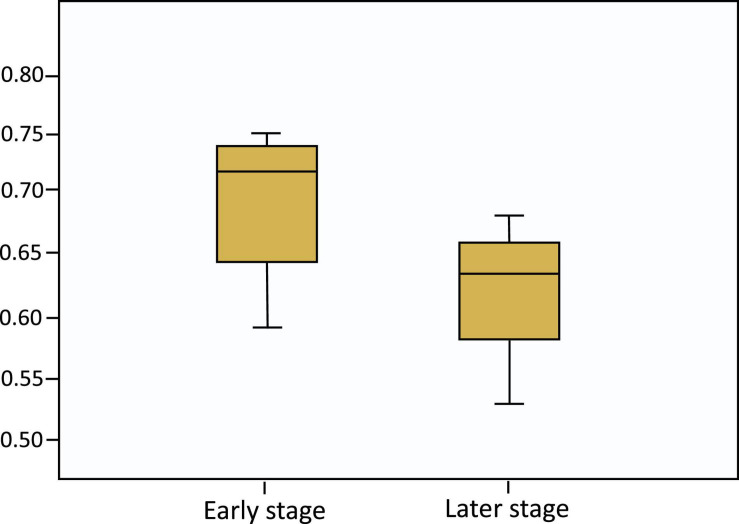
The distribution of network density of shared leadership in the early phase vs. later phase.

Led by the research of Farh et al. (2010), the phase of the project life cycle was measured from the percentage of the project work completed at the time of the survey, as reported by project managers. In the sample of our study, the mean project completion rate across 26 teams was 56%. This research checked journal guidelines and similar papers (see Farh et al., 2010) and used a mean split, where teams with a percentage of project completion equal to and below 56% were classified as being at an *early phase* and teams above 56% were classified as being at a *later phase*. Accordingly, there are 14 project teams in the early phase subgroup with the percentage of project completion ranging from 5% to 56%, and 12 in the later phase subgroup with 57–100% project completion. Figure 2 graphically illustrates the distribution of network density of shared leadership in the early phase vs. later phase.

#### Control Variables

Several control variables were included in the study. First is team size, as it has been proposed to be negatively related to the emergence of shared leadership (Cox et al., 2003) and negatively to customer ratings and team self-ratings of team effectiveness (Pearce and Sims, 2002). The second control variable is team tenure (the length of time an individual has worked on a specific team). It was included as it reflects the experience of group members working together which may influence team effectiveness (Marrone et al., 2007) and shared leadership because team longevity affects mutual familiarity, trust and interaction among team members (Cox et al., 2003). Third is team members’ educational levels, since the team member’s diversity has been demonstrated to moderate the relationship between shared leadership and team outcomes (Hoch, 2014). Therefore, team members’ educational levels were controlled, together with team size, team tenure for the analysis of this present research.

### Results

**FIGURE 3:**
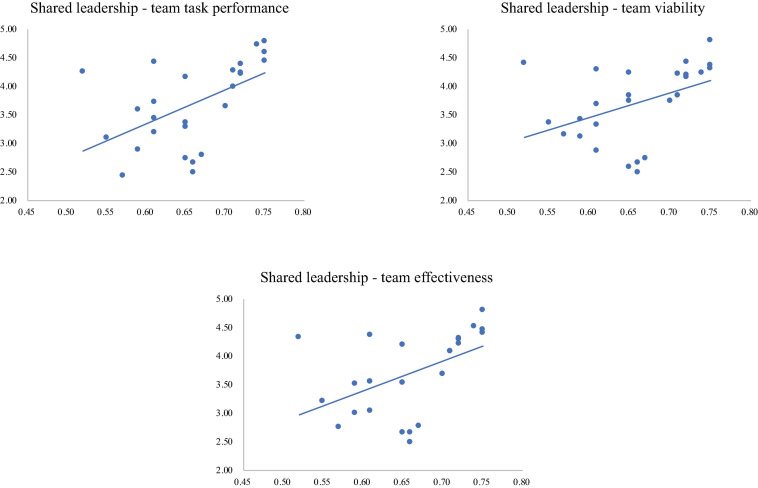
The three-panel correlation plot.

Table 3 presents the means, standard deviations and zero-order correlations of all the constructs. As illustrated, shared leadership is positively and significantly correlated to team task performance (*r* = 0.52, *p* < 0.01), team viability (*r* = 0.43, *p* < 0.05) as well as team effectiveness (*r* = 0.50, *p* < 0.05), which provides preliminary evidence to support hypothesis 1a, 1b, and 1c. Figure 3, a three-panel correlation plot, visually depicts the relationship between shared leadership and team task performance, team viability as well as team effectiveness.

**TABLE 3:** Descriptive statistics and correlations.

Variables	Mean	SD	1	2	3	4	5	6	7	8
1. Shared leadership	0.66	0.35	–							
2.Team task performance	3.69	0.74	0.53**	–						
3.Team viability	3.71	0.67	0.43*	0.92***	–					
4. Team effectiveness	3.70	0.69	0.50*	0.96***	0.97***	–				
5. Project life cycle	55.8	0.28	−0.46*	–0.38	–0.35	–0.37	–			
6. Team size	4.46	1.48	0.12	–0.09	0.11	–0.01	–0.17	–		
7. Team tenure	2.48	0.53	0.00	0.12	0.08	0.10	–0.02	0.03	–	
8. Educational diversity	2.19	0.20	–0.25	0.02	–0.05	–0.02	0.14	–0.02	0.07	–

*p < 0.05, **p < 0.01, and ***p < 0.001.

To further test the relationship between shared leadership and team effectiveness, as well as the moderating role of the project life cycle in such relationships, this research employed a two-way moderated hierarchical regression analysis (Carson et al., 2007; Erkutlu, 2012; Fausing et al., 2013). Led by the procedure delineated in Cohen et al. (2014), in the regression model, the control variables, team size, team tenure and educational diversity were entered in the first step for this research; shared leadership as an independent variable was entered in the second step; the interaction terms (predictor variable, shared leadership and moderator variable, project life cycle) was entered in the third step. In order to avoid multicollinearity problems, the standardized scores were utilized in the regression analysis (Aiken et al., 1991). Table 4 depicts the results of the moderated regression analyses.

**TABLE 4:** Results of regression analysis for team effectiveness.

	Team effectiveness		
Variables	Model 1	Model 2	Model 3
**Step 1**			
Team size	–0.01	–0.07	–0.11
Team tenure	0.10	0.09	0.10
Educational diversity	–0.03	0.10	–0.14
**Step 2**			
Shared leadership^a^		0.53*	0.26
**Step 3**			
Shared leadership × project life cycle			−0.47*
*R*^2^	0.10	0.27	0.41
*Adjust R*^2^	–0.13	0.13	0.26
*F*	0.08	1.95	2.76*

*p < 0.05.

**FIGURE 4:**
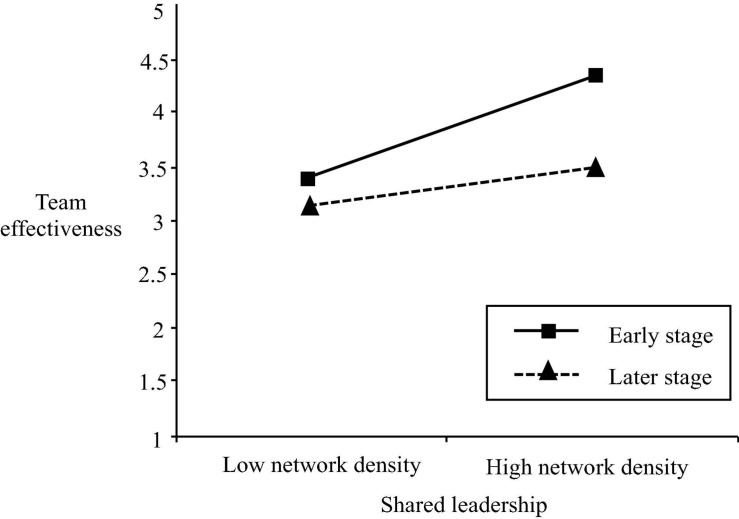
The moderating effect of the project life cycle on the relationship between shared leadership and team effectiveness.

As can be seen in step 1 in Table 4, the control variables were not significantly associated with team effectiveness. In step 2, we find that there is a significant positive relationship between shared leadership and team effectiveness (β = 0.53, *p* < 0.05), supporting hypothesis 1c (shared leadership is positively related to team effectiveness in engineering design teams). Moreover, the result of step 3 shows that the interaction between shared leadership and the project life cycle is significantly related to team effectiveness (β = −0.47, *p* < 0.05). We then graphically plotted the relationship between shared leadership and team effectiveness as moderated by the project life cycle (Figure 4) as recommended by Aiken et al. (1991). We see that a positive relationship is stronger in the early stage, when compared to the later phase of the project life cycle. Therefore, hypothesis 2 (the stage of project life cycle moderates the positive association between shared leadership and team effectiveness, such that this relationship will be stronger at the early phase than at the later phase of the project in engineering design teams) was fully supported in this study.

## Discussion

By integrating concepts from shared leadership, team effectiveness and project management literature, the current research sheds light on our understanding of *whether* and *when* shared leadership is positively related to team effectiveness. More specifically, this research advances prior work by demonstrating that there is a positive relationship between shared leadership and team effectiveness in Chinese engineering design teams. Furthermore, we also demonstrated that the stage of the project life cycle moderates the relationship between shared leadership and team effectiveness; where the positive association is stronger at the early phase than at the later phase of project life cycle. These findings provide significant theoretical contributions as well as practical implications.

### Theoretical Contribution

First of all, by joining a handful of researchers in the field of shared leadership (Liu et al., 2014; Chiu et al., 2016; Serban and Roberts, 2016), this study further confirms that shared leadership plays a significant role in building effective team outcomes. Specifically, this research linked shared leadership with team task performance [defined in terms of how well the group meets (or even exceeds) expectations regarding its assigned tasks]. Shared leadership has been consistently shown to be critical for improving team performance in practice and in the extant literature (Ensley et al., 2006; Carson et al., 2007; D’Innocenzo et al., 2014; Hoch, 2014; Wang et al., 2014; Chiu et al., 2016; Fransen et al., 2018). Although these studies have advocated the benefits of shared leadership on team performance, there is still some disagreement and controversy surrounding it (Mehra et al., 2006; Boies et al., 2011; Hmieleski et al., 2012). This current study therefore extends this line of research by demonstrating that the positive association between shared leadership and team task performance holds up in engineering design teams, thus supporting cogent work in the field of shared leadership. Moreover, the results of the current study also suggest that shared leadership is positively associated with team viability (considered in terms of the potential of teams to retain its members and to keep good team functioning over time). This finding is consistent with previous studies that suggested that shared leadership fosters team functioning and team member satisfaction. For example, Bergman et al. (2012) suggested that teams with shared leadership experience less conflict, greater consensus, and higher intragroup trust and cohesion than teams without shared leadership. Wood and Fields (2007) proposed that shared leadership exerts positive impacts on the job satisfaction of team members as shared leadership inherently advocates greater empowerment and autonomy. Therefore, as demonstrated in the current study, members of teams who share leadership, experience increased interdependence, higher levels of collaboration, and a greater sense of satisfaction. Furthermore, the ability to retain team members and to maintain positive team functioning over time is enhanced.

Another important theoretical contribution is that this study provides interesting insights into an important boundary condition of shared leadership effects. Specifically, this study investigated and demonstrated that phases of the project life cycle moderate the shared leadership-team effectiveness relationship; such relationship is stronger at the early phase than the later phase. The result of this investigation is consistent with the theory on the dynamic nature of shared leadership. As Avolio et al. (2009) noted, shared leadership is not a static, but a transferable and quite a fluid process, wherein roles and relations among individuals merge, co-evolve, and change throughout the entire life cycle of the project. Moreover, this result also supports the proposition proposed by Ford and Sullivan (2004) who asserted that creative ideas and strategies generated at the early stage of the team cycle are more likely to be valued and integrated into effective outcomes. Our findings extend this theory by identifying shared leadership as a potential source to encourage novel ideas. Specifically, at the early stage of the project life cycle where the focus is on planning and strategy generation, team members proactively participate in constructive communication and decision-making process. It thus provides a positive environment to nourish shared leadership. Such high-levels of leadership shared by individuals helps to generate more novel ideas, which could sequentially be valued and incorporated into effective results. Therefore, by integrating the project life cycle as a moderator, this study demonstrated how the temporal factor influence the shared leadership-team effectiveness association.

### Practical Lmplications

This research brings several significant practical implications to project management practitioners. Most notably, our findings confirm the positive relationship between shared leadership and team effectiveness in engineering design teams. It indicates that shared leadership can be a useful way to improve project team outcomes. This suggests that project managers seeking to foster high-levels of effectiveness should be supportive of sharing leadership within their groups and take steps to encourage group members to share leadership roles and responsibilities and provide them with adequate opportunities to interact with each other. Moreover, this study demonstrated that the association between shared leadership and team effectiveness is stronger at the early phase of the project life cycle. This emphasizes the need for managers to support shared leadership forms particularly at the early phase of the project in order to leverage benefits and maximize team effectiveness. Moreover, this research provides a benchmark with social network technique to help managers to assess their leadership development programs, in order to determine the extent to which they are reinforcing the notion of leadership as a collective process.

## Limitation and Future Research

As is the case for any research, there are some limitations related to this current study which are worthy of being acknowledged. First of all, since the measurements for the variables used in the study were taken from the same source, there could be common source bias influencing the relationship between shared leadership and team effectiveness. However, this research assessed team effectiveness by measuring the entire team’s behavior and outcomes, while shared leadership measured the behavior of individual members and was analyzed by a social network method. As such, the common source bias was mitigated to some extent because of this measurement distinction. In addition, the sample of this experimental study consisted of 26 teams for both the early and later phase of the project life cycle. Replications of current research and future studies are encouraged to increase the sample size so as to achieve greater statistical power.

Second, while the definition of team effectiveness (measured in terms of team task performance and team viability) is multidimensional in nature, it does not take every possible aspect into consideration, e.g., happiness of the team members. In other words, the predictors used in this research are not an exhaustive list. There can be other consequences of shared leadership that have not been accounted for. This study thus encourages more studies to examine additional predictors of shared leadership, especially predictors from a multilevel perspective. For example, more consequences at the firm and organizational level should be examined, e.g., firm competitive advantage, organizational effectiveness and creativity. Furthermore, since our research focused only on engineering design teams, it limits the generalizability of the results. Therefore, future studies can make a valuable contribution by examining the relationship between shared leadership and its outcomes from a wide variety of contexts.

Third, an important premise of this investigation, regarding when shared leadership influences team effectiveness across the project life cycle, is the dynamic nature of shared leadership. Its emergence is likely to be influenced by team environments (i.e., cross-functional communication and coordination, and active participation in the decision-making process); as well as task characteristics (i.e., creative tasks). Unfortunately, the design of the current study did not directly examine these factors that could simulate the occurrence and development of shared leadership. It thus would be a promising research direction for future studies. Moreover, since shared leadership is a dynamic and emergent process, research with a longitudinal design that captures multiple iterations and cyclic feedback loops of shared leadership, to understand how it changes or evolves throughout stages of the project team life cycle, is another fruitful avenue for future studies.

Fourth, this study is among the first to explore the moderating role of the project life cycle in the relationship between shared leadership and team effectiveness. We thus encourage future research to provide a more complete understanding of the boundary conditions of shared leadership effectiveness, particularly for project-related moderators. Examples like project complexity, project uncertainty, and project creativity are worthy of attention in future studies. Moreover, the potential temporal indicators should also be examined considering shared leadership is a dynamic process in nature. This would serve as another promising direction for future research.

Fifth, shared leadership, as a new leadership pattern that has been demonstrated to facilitate team effectiveness in the engineering design teams. However, we do not advocate that shared leadership is a panacea for all organizational woes. There may be many circumstances where shared leadership is not suitable e.g., non-knowledge teams. Furthermore, Pearce (2004) suggested that shared leadership is a more complex and time-consuming process than traditional vertical leadership. In light of this, research concerning when and for whom shared leadership is inappropriate should be another interesting avenue and thus worthy of further attention.

## Contribution

The current study was designed to produce novel theoretical and empirical insights regarding *whether* shared leadership is positively related to team effectiveness and *when* shared leadership is more likely to be effective. By demonstrating a positive association between shared leadership and team effectiveness in engineering design teams, this study adds to a growing literature extolling the value of shared leadership. Another important contribution of the present research is that it is among the first to investigate a temporally relevant moderator, the project life cycle, for the effectiveness of shared leadership. The authors hope that the insightful findings gained through this effect will spur future studies aimed at understanding the dynamics of shared leadership in project teams and further explore temporal factors for its effectiveness.

## Data Availability Statement

The raw data supporting the conclusions of this article will be made available by the authors, without undue reservation.

## Ethics Statement

The studies involving human participants were reviewed and approved by Graduate Research Committee (GRC), National University of Ireland, Galway. The patients/participants provided their written informed consent to participate in this study.

## Author Contributions

QW was responsible for conducting analysis and writing the first draft. KC contributed to the structure and content and revised all versions of the manuscript. QW and KC both participated in idea development.

## Conflict of Interest

The authors declare that the research was conducted in the absence of any commercial or financial relationships that could be construed as a potential conflict of interest.
